# Evaluation of Small Molecule Drug Uptake in Patient-Derived Prostate Cancer Explants by Mass Spectrometry

**DOI:** 10.1038/s41598-019-51549-3

**Published:** 2019-10-18

**Authors:** Shadrack M. Mutuku, Paul J. Trim, Bala K. Prabhala, Swati Irani, Kayla L. Bremert, Jessica M. Logan, Douglas A. Brooks, Jürgen Stahl, Margaret M. Centenera, Marten F. Snel, Lisa M. Butler

**Affiliations:** 1 Adelaide Medical School, University of Adelaide, Adelaide, SA 5005 Australia; 2 Prostate Cancer Research Group, South Australian Health and Medical Research Institute (SAHMRI), Adelaide, SA 5000 Australia; 3 South Australian Health and Medical Research Institute (SAHMRI), Adelaide, SA 5000 Australia; 4 Department of Drug Design and Pharmacology, University of Copenhagen, København, Denmark; 5 Freemasons Foundation Centre for Men’s Health, University of Adelaide, Adelaide, SA 5005 Australia; 6 Mechanisms in Cell Biology and Disease Research Group, School of Pharmacy and Medical Sciences, Cancer Research Institute, University of South Australia, Adelaide, SA 5000 Australia; 7 Clinpath Laboratories, Adelaide, SA 5000 Australia

**Keywords:** Drug delivery, Cancer

## Abstract

Patient-derived explant (PDE) culture of solid tumors is increasingly being applied to preclinical evaluation of novel therapeutics and for biomarker discovery. In this technique, treatments are added to culture medium and penetrate the tissue via a gelatin sponge scaffold. However, the penetration profile and final concentrations of small molecule drugs achieved have not been determined to date. Here, we determined the extent of absorption of the clinical androgen receptor antagonist, enzalutamide, into prostate PDEs, using liquid chromatography-tandem mass spectrometry (LC-MS/MS) and matrix-assisted laser/desorption ionisation (MALDI) mass spectrometry imaging (MSI). In a cohort of 11 PDE tissues from eight individual patients, LC-MS/MS quantification of PDE homogenates confirmed enzalutamide (10 µM) uptake by all PDEs, which reached maximal average tissue concentration of 0.24–0.50 ng/µg protein after 48 h culture. Time dependent uptake of enzalutamide (50 µM) in PDEs was visualized using MALDI MSI over 24–48 h, with complete penetration throughout tissues evident by 6 h of culture. Drug signal intensity was not homogeneous throughout the tissues but had areas of markedly high signal that corresponded to drug target (androgen receptor)-rich epithelial regions of tissue. In conclusion, application of MS-based drug quantification and visualization in PDEs, and potentially other 3-dimensional model systems, can provide a more robust basis for experimental study design and interpretation of pharmacodynamic data.

## Introduction

Prostate cancer is the most commonly diagnosed male cancer and the second leading cause of cancer deaths in Western countries^1^. Androgens and their intracellular target, the androgen receptor (AR), are established drivers of prostate cancer initiation, development and progression^2,3^. As such, blocking androgen action by androgen deprivation therapies or by selective inhibition of the AR with antagonists has been the mainstay of past and current therapeutic interventions for prostate cancer^4^. However, these strategies are not curative and durable tumor responses are currently unattainable^5^. Enzalutamide (ENZ) is a second-generation AR antagonist with additional modes of action compared to first-generation agents, namely suppression of AR nuclear translocation and binding of the androgen-AR complex to DNA^5^. Enzalutamide is currently used for treatment of metastatic castration resistant prostate cancer (CRPC)^6,7^, and was recently approved for men with high-risk localized disease (HRLD)^3,8,9^. Unfortunately, enzalutamide treatment is not curative, and intrinsic and acquired resistance are both common, representing critical research priorities in the field^3^. There is an increasing appreciation that using patient-derived tumor models may reveal more clinically-relevant mechanisms of drug activity and/or resistance^10^. However, the interpretation of drug-related endpoints in such models requires an understanding of the penetration of small molecule agents into the tissue structure, and the in-tissue drug concentrations achieved during culture.

Mass spectrometry is used extensively in the characterisation of pharmacokinetic behaviour of compounds in drug development^11^, for quantification of metabolites^12^ and hormones^13^. Orthodox quantification assays typically involve homogenization of the drug-containing tissue or biofluid, sample clean-up, and separation by liquid chromatography followed by absolute quantification using tandem mass spectrometry (LC-MS/MS). Although such approaches yield accurate quantification data, homogenization of tissue samples results in complete loss of spatial information and all measurements represent integrated values for the whole tissue, thereby making it impossible to know whether the molecule of interest is evenly distributed or differentially bio-accumulated, dependent on cell type and tissue penetration. With the advent of mass spectrometry imaging (MSI), it is possible to retain spatial information, allowing detailed mapping of the tissue distribution of molecules of interest such as drugs, metabolites or endogenous compounds such as phospholipids^14–16^. MALDI MSI involves laser-desorption/ionization of molecules from a thin layer matrix-coated biological sample on a glass slide^17,18^. An intensity map can be generated to visualize and quantify specific ions based on their *m/z*, resulting in a form of chemical histology^19^. MSI currently has two broad applications based on target molecular mass. Large molecule imaging typically encompasses *in situ* proteomic profiling, revealing protein and peptide composition^16,20^, while small molecule imaging has largely characterized metabolites such as sugars, fatty acids^21^ and lipids^19,22^. Many pharmacological small molecule agents occupy a similar chemical space (comparable molecular weights, physicochemical properties and functional groups) as endogenous metabolites. Consequently, drug imaging in preclinical and clinical settings constitutes an important emerging area of small molecule imaging^23^.

Determination of enzalutamide quantities in human plasma and rat plasma for pharmacokinetics studies has been previously described^24,25^ but has not been attempted in a tissue culture model. Herein, we utilized LC-MS/MS and MALDI MSI to both quantify and visualize the profile of enzalutamide uptake from culture in an ex *vivo* PDE model of clinical prostate cancer. Moreover, we describe the spatial localization of enzalutamide-derived ion signals in tissues with heterogenous histological features and in comparison to immunohistochemical staining of the intracellular drug target, AR.

## Results

### Enzalutamide is detectable in Patient-Derived Explant (PDE) culture systems

**Figure 1:**
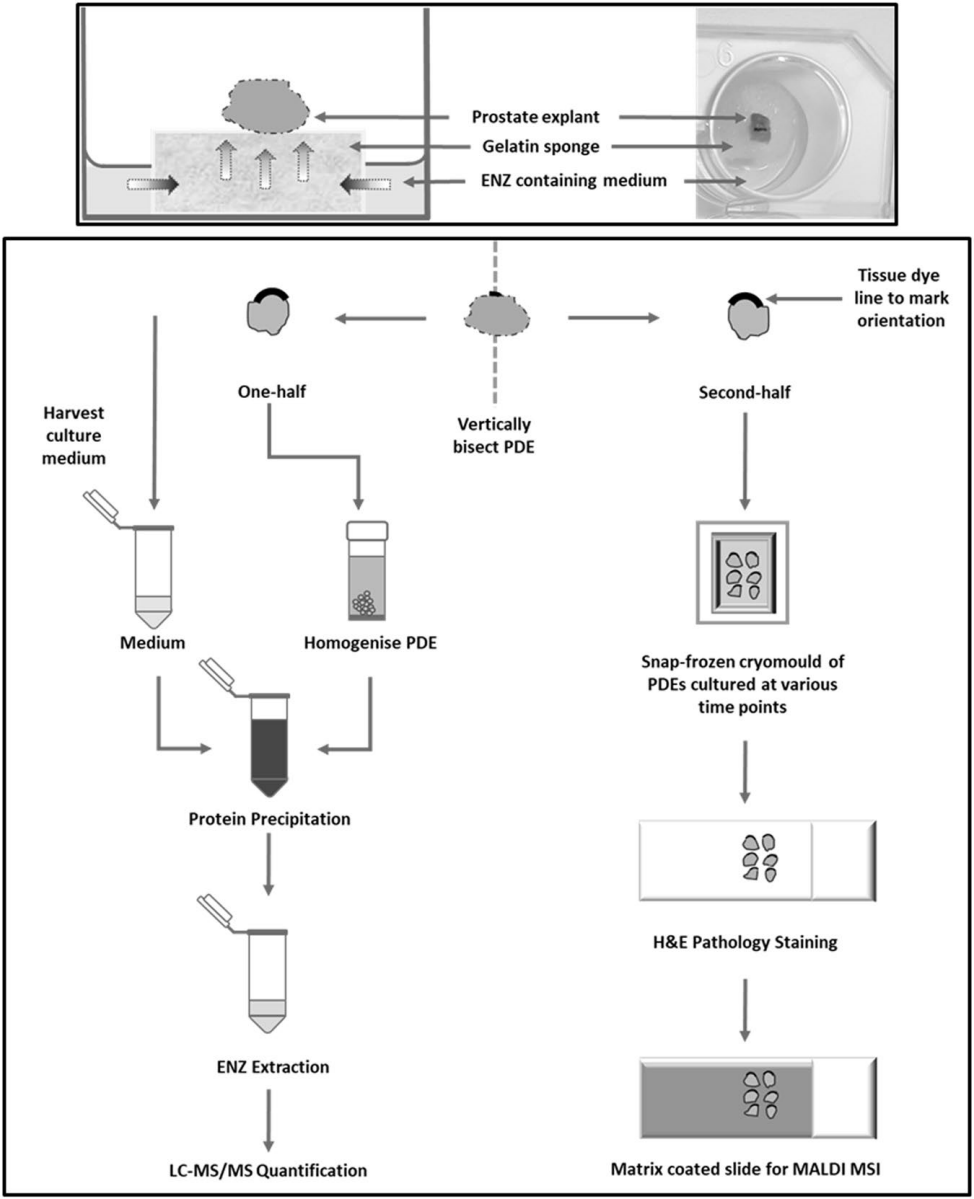
Schematic of drug uptake in *ex vivo* culture of human prostate tumors. Top panel. Schematic and photograph of a PDE on top of a gelatine sponge in a well with 500 µL medium. Bottom panel. Workflow chart for evaluation of ENZ uptake in human prostate PDEs by mass spectrometry.

**Figure 2:**
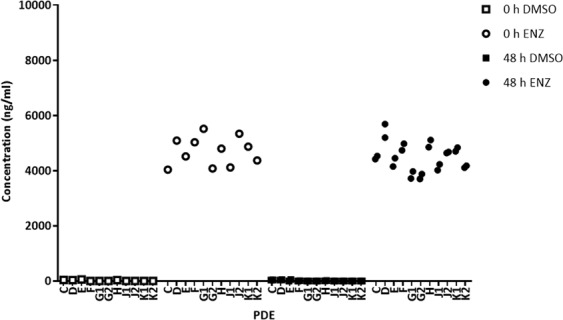
LC-MS/MS quantification of ENZ in conditioned explant media. PDE’s were cultured in medium containing 10 µM ENZ or DMSO control over a 48h period. LC-MS/MS quantification of the predominant ENZ fragment ion, *m/z* 209.09 is shown. Drug concentration in medium expressed as ng/ml.

We developed, and validated according to US FDA guidelines, a quantitative LC-MS/MS assay capable of measuring enzalutamide levels in both culture media and tissue specimens from our widely used PDE model system^26,27^ (Fig. 1). In a cohort of 11 prostate tissues from 8 patients (3 patients provided cores from the left and right sides of the prostate), enzalutamide (ENZ) was readily detectable above the lower limit of quantitation (LLOQ) in conditioned medium after 48 h of culture with a working dose of 10 µM (4.6 µg/ml), and concentrations were within 15% of assay quality control (QC) measurements (Fig. 2, Supplementary Table ST2). Enzalutamide was not detected in medium from vehicle control (DMSO) wells, confirming assay selectivity. For each patient sample, enzalutamide concentration values in media were highly consistent when comparing 0 h versus 48 h of culture (Fig. 2), indicating that the drug remained stable in the medium throughout the culture period.

Next, the concentration of enzalutamide was assessed in conditioned medium from PDEs cultured at two different concentrations of ENZ (10 µM or 50 µM) for time points up to 24 h. Stability of ENZ at both concentrations was evident over the 24 h incubation period (Table 1), while control samples showed nil amounts of drug present in the well at all time points. The integrity of enzalutamide in the cryo-stored samples (−80 °C) was confirmed by including freshly prepared, dilution quality control (DQC), samples at the same concentration as the experimental test samples.

**Table 1:** LC-MS/MS quantification of 10 µM^ and 50 µM ENZ* in culture media.

Concentration (ng/ml)	Patient A-L^	Patient A-R^	Patient B-L*	Patient B-R*
DMSO Stability	—	—	0	0
DMSO 0 h	0	0	0	0
DMSO 24 h	0	0	0	0
DQC	4410	4410	23170	23170
DS well	—	—	26200	26200
ENZ 0 h	4620	4220	23600	26100
ENZ 1 h	4700	4420	21700	20200
ENZ 2 h	4070	4280	20500	19300
ENZ 6 h	3450	4560	18500	20100
ENZ 24 h	4130	4470	19900	19100

Six PDEs from separate prostate cores left (L) and right (R) of two patients, A and B, were treated at indicated dose of ENZ for 1 h, 2 h, 6 h, 24 h or 24 h control; media from corresponding wells was collected by snap-freezing at respective time points including 0 h and 24 h control. DQC values are an average of triplicate measurements. Values are expressed as ng/ml.

**Figure 3:**
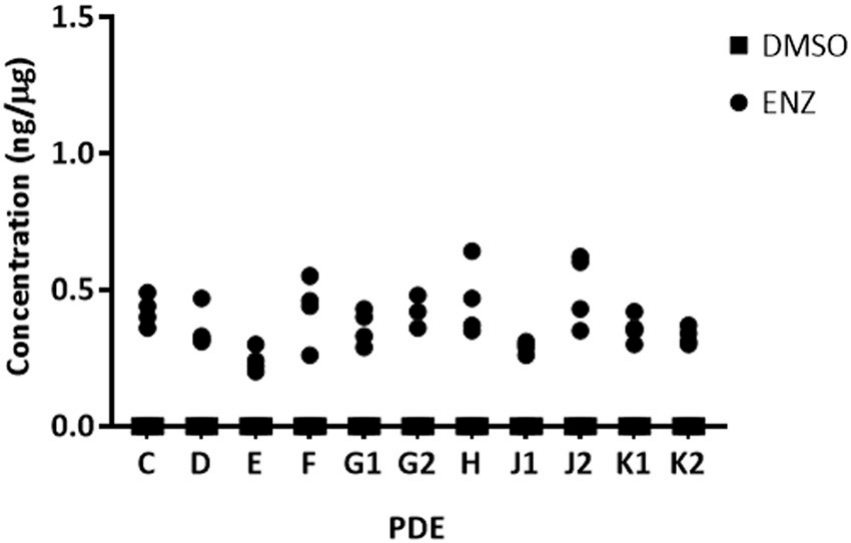
LC-MS/MS quantification of ENZ in PDE homogenates. PDEs were cultured in medium containing 10 µM ENZ or DMSO control over a 48h period. LC-MS/MS quantification of the ENZ MRM transition 465.1 → 209.09 is shown. Measured drug concentration in homogenate is normalized to total protein expressed as ng/µg of total protein. Intra-patient variability of 17.7% and intra-patient variability of 20.1%. n = 4 (two explant pieces per well containing medium with 10 µM ENZ or DMSO vehicle).

For assessment of ENZ uptake in PDE tissues, initially quadruplicate pieces of tissue were analyzed for each PDE sample (n = 11) after 48 h of treatment at 10 µM. ENZ was detected above the LLOQ in all homogenates. While disparity in ENZ content was evident between tissues and patients, the concentrations achieved were comparable in magnitude, 0.24–0.50 ng/µg of total protein. The drug absorption exhibited an intra- and inter-patient variability of 17.7% and 20.1%, respectively (Fig. 3, Supplementary Table ST3). The major active metabolite of ENZ, *N*-desmethylenzalutamide, was not detectable in any PDE homogenates.

### Visualization of enzalutamide uptake in prostate PDE tissues

The spatial kinetics and distribution of ENZ uptake in *ex vivo* cultured PDEs was visualized by MALDI MS/MS imaging of the predominant ENZ fragment ion, *m/z* 209.09. In a pilot study, the penetration of ENZ (50 µM) was assessed in frozen PDE sections cultured for defined times (1, 5, 20 or 48 h). Uptake of drug into the tissues was evident and extended throughout the PDEs by 5 h of culture, confirmed by imaging multiple depth planes in the tissues (Supplementary Fig. S1). The fragment ion of the drug then remained detectable at similar intensity over the remaining incubation period (Supplementary Fig. S1). Whereas it was possible to map the intensity distribution of ENZ signal by MALDI MS/MS imaging in PDEs cultured at both 10 and 50 µM doses, the relative abundance of *m/z* 209.09 from the 10 µM treatment generated insufficient signal intensity compared to the 50 µM treatment for reliable data generation. This was confirmed by LC-MS/MS quantification of tissue from 10 µm sections in a subset of PDEs, where the level of ENZ quantified at 10 µM was at the limit of detection (LOD) and 50 µM was above the lower limit of quantification (LLOQ) (Supplementary Fig. S2). Accordingly, 50 µM of ENZ was selected for further MALDI MSI studies.

### MALDI MSI and LC-MS/MS indicate the kinetics of drug uptake in PDEs

**Figure 4:**
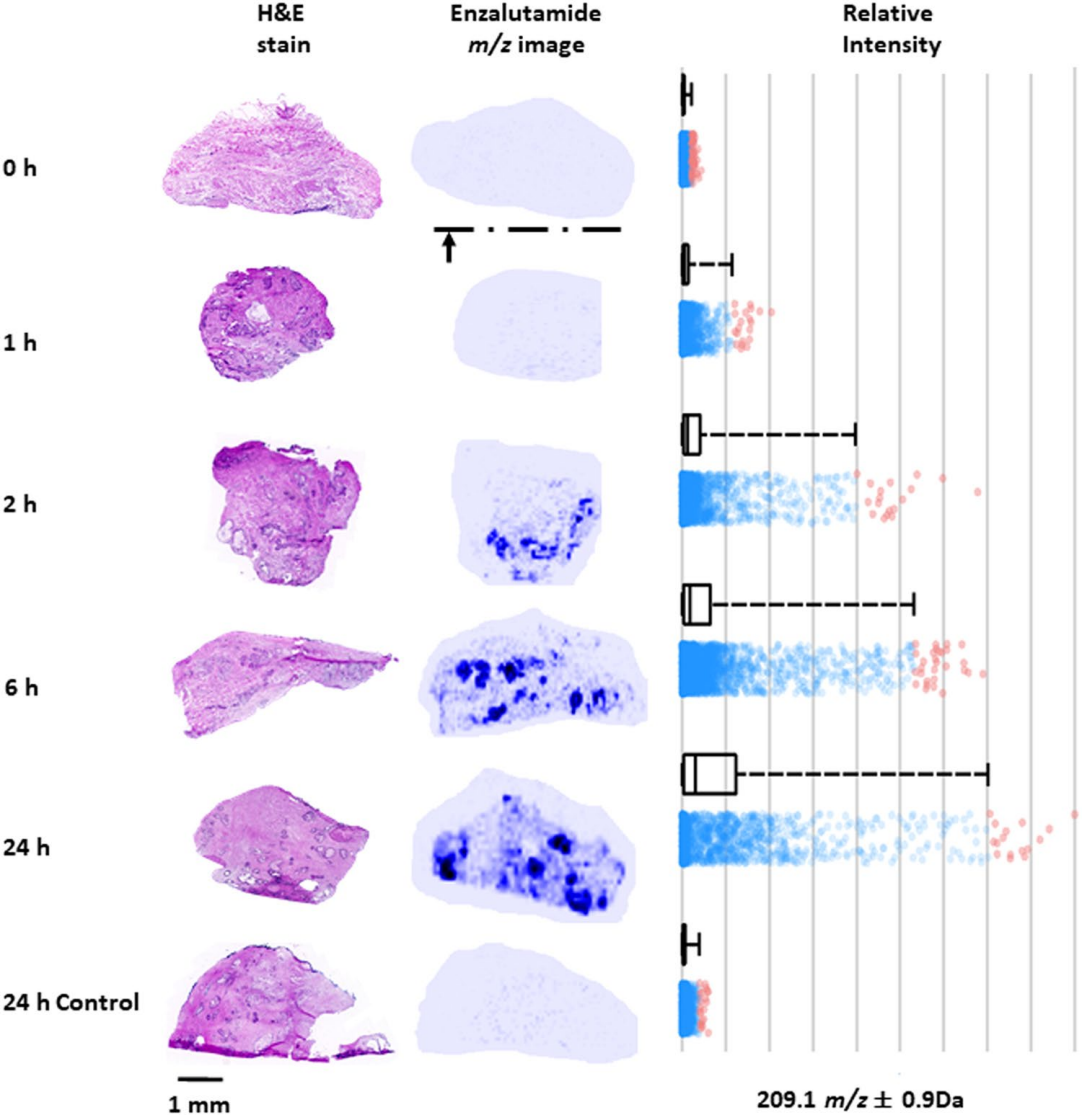
Time profile of ENZ penetration in *ex vivo* prostate tissue culture. Left panel. Six PDE’s were cultured in medium containing 50 µM ENZ over a 24 h period or DMSO control (24 h). MALDI MSI MS/MS images of the predominant ENZ fragment ion, *m/z* 209.09, are shown with normalization to TIC next to a serial section stained by H&E. The arrow at the bottom of the image at 0 h indicates the contact of the PDE with the gelatin sponge surface. H&E sections are 30 µm adjacent to the MALDI image tissue section. Right panel. Relative intensity of *m/z* 209.09 at 0 h, 1 h, 2 h, 6, h and 24 h/24 h control of respective MS/MS images (A) show a time-dependent increase in drug concentration in the tissues during culture. Red dots represent the spectra of enzalutamide outside of the upper quartiles.

Next, the spatial profile of ENZ uptake was assessed over a 24 h incubation period using 50 µM ENZ treatment in two independent PDEs from another patient. Data for the left core of the prostate in a single patient is shown (Fig. 4). For this experiment, the images of the sections correspond to the planar orientation of the explant such that the bottom of each tissue image indicates the site of contact between the explant and the gelatine sponges in the well (Fig. 1). Cumulative uptake of ENZ from the sponge surface into the PDEs was detected over the 24 h period, and confirmed by quantification of ion signal intensity (Fig. 4). At time 0 h there was no drug signal detected in the tissue. After 1 h of incubation, ENZ was evident at low levels, primarily at the bottom (sponge-contacting) edge of the PDE, and by 2 h the drug was abundant in the lower areas of the tissue. By 6 h–24 h, there was distribution evident throughout the tissue. The 24 h control treatment had no signal from the ENZ fragment ion, *m/z* 209.09, similar to the 0 h uncultured tissue (Fig. 4, Supplementary Fig. 1).

**Figure 5:**
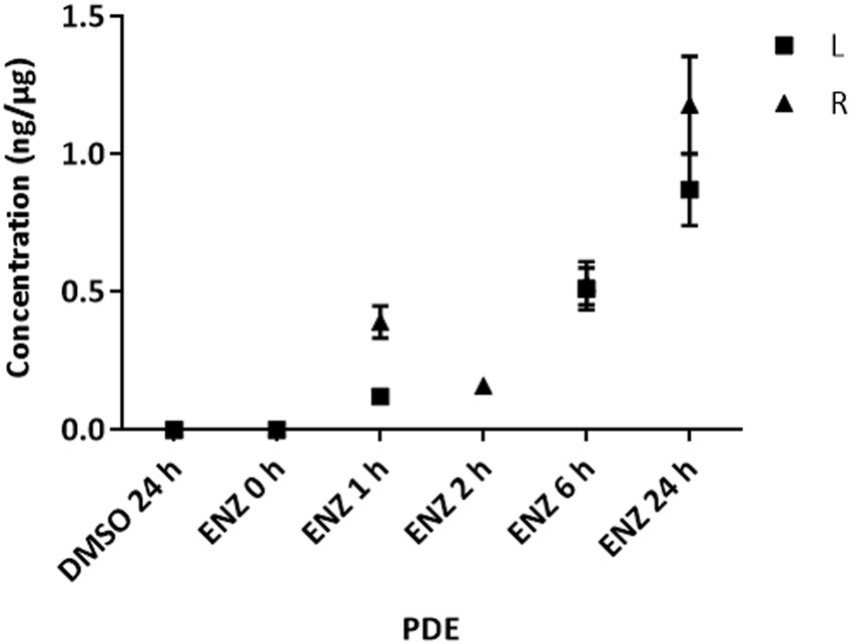
LC-MS/MS quantification of ENZ in PDE homogenates over a time course. Six PDEs from either the left (L) and right (R) prostate cores were cultured in 50 µM ENZ and harvested at 0 h, 1 h, 2 h, 6 h, 24 h or 24 h (DMSO control) and bisected. One-half was homogenized for LC-MS/MS analysis. Drug concentration is normalized to total protein expressed as ng/µg. *Note: PDE for L at 2 h lost during sample homogenization. Error bars indicate the 15% CV threshold for accuracy and precision of assay.

In parallel, the amount of ENZ absorbed by PDEs was quantified by LC-MS/MS on the bisected tissue halves of each of the treated PDEs from Fig. 4. This confirmed a time-dependent increase in tissue drug concentrations (Fig. 5, Supplementary Fig. S3). The maximum drug concentration reached after 24 h of culture following 50 µM dose was 0.87–1.18 ng/µg of total protein. Again, the *N*-desmethylenzalutamide metabolite fragment ion of ENZ (*m/z* 201.1) was undetectable.

### Enzalutamide accumulates in epithelium-rich regions of prostate tissue

**Figure 6:**
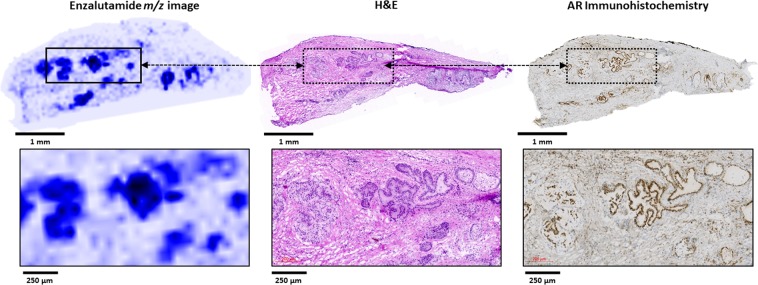
ENZ signal co-localization in prostate tissue epithelium. Comparison of the MS/MS image (ENZ fragment ion, *m/z* 209.09, left) to an H&E scan (middle) and immunohistochemical detection of the androgen receptor (AR). Lower panels: higher magnification images.

Notably, the pattern of enzalutamide signal detected by MALDI MSI in the prostate tissues was not homogenous throughout the tissue sections, but invariably displayed discrete regions of high signal. Comparison of serial sections of MALDI MS/MS images to the tissue histology (H&E staining) revealed that areas of high enzalutamide signal co-localized with histological tissue regions containing high epithelial cell content. (Fig. 6). The co-location of ENZ ion signal with benign or malignant prostate epithelial cells was further confirmed by immunohistochemical staining for AR in consecutive adjacent sections (Fig. 6).

## Discussion

There is increasing interest in applying patient-derived tissue model systems such as explants and/or xenografts to preclinical cancer research and drug development^26–28^. Such systems maintain 3-dimensional tumor structure, which in turn retains important features such as hormone responsiveness in the case of breast and prostate cancers^26^. A potential advantage of PDEs is the ability to relate drug responses to spatial features of the tissues, thereby allowing consideration of tumor multifocality and histological features of the tumor microenvironment. However, a key knowledge gap has been the accurate measurement of the degree and spatial kinetics of tissue penetration of such models by small molecules, and the effective intra-tissue concentrations achieved. This is the first study to both quantify and visualize uptake of a current clinical agent, enzalutamide, into clinical PDEs during culture. Moreover, by incorporating tissue histology and immunostaining we demonstrate marked accumulation of enzalutamide in the tissue epithelium, which highly expresses the agent’s intracellular target, the AR^3^.

MSI of non-endogenous small compounds has been explored previously in the study of anti-cancer agents in solid tumors, but this is largely confined to *in vivo* animal models. The distribution of the anti-cancer drug vinblastine has been directly performed on rat whole body sections by MALDI MSI^29^. Kwon and colleagues have reviewed the uptake of various anticancer agents in multiple solid tumors^30^. They reported localization of the protein kinase inhibitor vemurafenib within tumor regions of human malignant melanoma, where it specifically bound to its targets, BRAF mutated proteins, as opposed to BRAF negative tumor regions. MALDI MSI was also used to elucidate pharmacodynamic/pharmacokinetic properties of the anti-angiogenic small molecules and their metabolites, YCG185 and sunitinib, both targets for VEGFR2^30^. Specifically, for sunitinib, they demonstrated downregulation of the target receptor in response to treatment in a murine model of colorectal cancer. Thus, MALDI MSI supplemented with immunohistochemistry approaches is a powerful and spatially informative tool to assess drug-target engagement in preclinical studies. MSI of drugs and their metabolites has also been investigated in whole-body murine models^31,32^. This platform offers a unique advantage of observing drug distribution within pathophysiological areas of organ^33^ and tissue microenvironments^33^. The pharmaceutical industry has adopted whole body autoradiography (WBA)^34^ and MALDI MSI for drug imaging, although this has not been as extensive in academic research. Although WBA is a robustly quantitative and sensitive technique, it requires the use of expensive radiolabelled isotopes that yield indistinguishable organ images of the drug analytes from its metabolites of interest. However, MALDI MSI can be used for the direct analysis of a drug and can employ more relatively affordable deuterated analogues that can be spatially resolved from metabolites with high molecular specificity.

Here, we combined MALDI MSI with a highly sensitive LC-MS/MS assay to assess stability of enzalutamide in explant culture conditions and its uptake and distribution in the tumor tissues. These quantitative results indicate the maintenance of drug stability in PDE culture medium as a biological matrix over the entire culture period, and ensuring its availability in desired concentrations to perfuse viable tissue. The variability in comparative intra-patient drug absorption (measured amount of drug between replicate explants dissected from the same biopsy core) was evident. Nevertheless, the inter-patient drug uptake was still consistent in a diverse range of patient samples including benign tissue or heterogenous Gleason grades when normalized to total protein content of the tissues. Our results suggests that is unlikely that the variation in clinical response to enzalutamide is related to its uptake and the clinical features of the tissues. Recently, others have argued at the cellular level that during tumorigenesis prostate cancer cells produce altered lipid membranes^35,36^, which may reduce the efficacy of enzalutamide and lead to treatment resistance^35^. Given that our targeted MALDI MSI revealed marked accumulation of enzalutamide in AR-expressing epithelial cells, the positivity and/or expression level of the AR is likely to be another important determinant of enzalutamide content in individual tissues.

The recommended clinical dose of enzalutamide is 160 mg orally once daily for treatment of metastatic CRPC^37^. The dosage of enzalutamide employed in our *ex vivo* model of human prostate cancer PDE model is 10 µM (4.6 µg/ml) and importantly, this is comparable to plasma concentrations readily achieved systemically in clinical patients^38^. It is consistent with the clinical dose described by Gibbons and colleagues who analyzed the pharmacokinetics of enzalutamide and *N*-desmethylenzalutamide from five clinical studies^39^. They also reported after a consecutive multiple oral doses of 160 mg that the pre-dose steady-state mean plasma concentration (*C*_trough_) reached was 11.4 µg/ml^39^. Moreover, 10 µM enzalutamide is a routinely employed dosage in pre-clinical models, both cell culture^40^ and *in vivo* studies^41^, of prostate cancer for evaluation of its efficacy in combination with other non-AR targeted genome therapies^41–43^. PDEs often require 5–10-fold higher drug doses than in 2-dimensional cell culture studies to attain equivalent efficacy and the 10 µM dose was primarily evaluated in this study.

MALDI MS/MS imaging experiments of 10 µm sections of PDES treated with 10 µM enzalutamide yielded low signal intensity close to the limit of detection and consequently the spatial drug visibility during the 24 h time profile was unreliable. Using targeted MALDI MS/MS, we have evaluated a higher dose of 50 µM (~25 µg/ml) enzalutamide that approximates to 360 mg/day administered as multiple oral doses previously reported^38^. The higher dose provided high quality MSI data, and allowed us to ascertain the drug-target engagement by MSI and revealed its high affinity for epithelial cells that are known to express its intracellular target receptor, AR. The time-dependent increase in *in situ* drug concentration we observed may explain the drugs’ reported clinical pharmacokinetic parameters of a half-life of 5.8 days and accumulating at least 8-fold^39^. The major active metabolite, *N*-desmethylenzalutamide was not detected, likely because enzalutamide primarily undergoes hepatic metabolism^38^.

In conclusion, understanding the parameters of agent penetration and accumulation is essential to undertake pharmacodynamic experiments using PDE preclinical model systems. Using a mass spectrometry-based experimental approach, we demonstrate for the first time that the current clinical agent enzalutamide fully and rapidly suffuses cultured prostate tissues, thereby identifying optimal endpoints for experimental assays involving ENZ. More broadly, such approaches warrant inclusion in translational research involving not only PDEs but other 3-dimensional models systems such as patient-derived organoids, where penetration rates of various small molecular agents will profoundly influence both study design and interpretation of experimental endpoints.

## Methods

### Chemicals

α-cyano-hydroxycinnamic acid (CHCA), polyethylene glycol (PEG) standards (200, 600, 1000) were obtained from Sigma-Aldrich. Carboxymethylcellulose (CMC) and dimethlysulfoxide (DMSO) were purchased from Sigma-Aldrich (NSW, Australia). Methanol and acetonitrile (all LC-MS hyper grade, Lichrosolv) were purchased from Merck (VIC, Australia). Argon (Catalogue No. 262) for mass spectrometry application was purchased from BOC (NSW, Australia). Formic acid was purchased from Fisher Chemicals (St. Louis, MO, USA). RPMI 1640 phenol free culture medium (Catalogue No. 1185-030) was purchased from Life technologies (USA). Gelatin sponges were purchased from Johnson and Johnson (NC, USA). Water was obtained from a MilliQ system with 0.22 µm filter. Micro Bicinchoninic acid (BCA Catalogue No. 23235) assay kit was purchased from Thermo Scientific (Rockford, IL, USA). Enzalutamide (ENZ) (MDV3100 Catalogue No. S1250 000-09083) was purchased from Selleckchem (Houston, TX, USA). [^2^H_6_]-enzalutamide and [^2^H_6_]-desmethylenzalutamide were purchased from Alsachim (Strasbourg, France). Chemical structures are shown in Supplementary Fig. S4.

### *Ex Vivo* culture of human prostate tissue

We employed a well-defined explant model of patient-derived prostate tumor explants (PDEs)^26,27^. Following radical prostatectomy, 6–8 mm tissue cores from the left and/or right sides of the prostate were dissected into approximately 1–3 mm^3^ pieces and cultured in quadruplicate, as described previously^26^. The pathological information for n = 8 patients (yielding 11 separate tissue samples), indicating Gleason grade, clinical stage and prostate specific antigen (PSA) levels, as well as rescored pathology (J.S.) for the PDE-derived tissue samples, is shown in Supplementary Table ST1. For measurement of homogenates, PDEs were cultured for 48 h in the presence and absence of 10 µM ENZ, with samples collected at 0 h and 48 h (Fig. 1). For time course experiments tissues were cultured in the presence or absence of 10 or 50 µM ENZ and samples were harvested at a range of time points as described. Controls included both untreated fresh frozen tissue and DMSO at 24 h, as well as a drug stability (DS) well that included gelatine sponge and medium only without tissue, and a drug quantification (DQ) well of gelatine sponge, medium and explants. The DS well monitored the integrity of ENZ during incubation period.

### LC-MS/MS

The LC-MS/MS method was validated according to US FDA guidelines prior to sample analyses^44^. ENZ was extracted from the culture media, snap-frozen PDE tissues and/or PDE frozen tissue sections. Briefly, 250 µL of 1:100 diluted medium was spiked with 50 µL of 1 µM deuterated (^2^H_6_) ENZ and 50 µL ^2^H_6_ desmethylENZ internal standard (IS) and subjected to protein precipitation using pre-chilled acetonitrile (1:4 v/v) and incubated at −20 °C for 1 h. Samples were then centrifuged at ca 16,000 *rcf* at 4 °C for 20 min. The supernatant was aliquoted into new tubes, freeze-dried and reconstituted to starting volume of 400 µL with methanol-water 1:1 (v/v). PDE samples from each time point were placed in 2.0 ml tough tubes (Mo Bio Laboratories, CA, USA) with 1,000 µl methanol-water 1:1 (v/v) for homogenisation using a Precellys 24 homogeniser (Bertin Technologies, Australia) set at 6500 rpm for 30 sec twice with a 30 sec rest period in between.

Total protein in tissue homogenates was measured using the Micro BCA protein assay kit following manufacturer’s protocol. 250 µl of PDE tissue homogenates were subjected to the same protein precipitation and drug extraction procedure as described above.

LC-MS/MS analysis was performed on an Acquity ultra-performance liquid chromatography (UPLC) system (Waters Corporation, MA, USA) coupled to an API 4000 QTrap (Applied Biosystems MDS Sciex, Ontario, Canada). Chromatography employed a BEH C18 1.7 μm particle size 2.1 mm by 50 mm column (Waters, Corporation, Ireland). Solvent A was 0.1% aqueous formic acid and solvent B was 99.9% acetonitrile with 0.1% formic acid. Other conditions were; column temperature, 30.0 °C, sample temperature, 6.0 °C and total run time, 7.40 min. An injection volume of 5 µL of reconstituted analyte was used and samples were eluted using a solvent gradient of 1–99% B from 0.2 min to 4.0 min, the column was washed from 4.0 min to 5.8 min with 99% B and re-equilibrated between 5.81 min to 7.40 min at 1% B at 0.35 ml/min flow rate. The mass spectrometer was operated in multiple reaction monitoring (MRM) mode. Transitions of *m/z* 465.1 to 209.2 (ENZ), *m/z* 471.1 to 215.1 ([^2^H_6_]-ENZ), *m/z* 451.1 to 201.1 (desmethylENZ) and *m/z* 457.1 to 195.1 ([^2^H_6_] – desmethylENZ) were monitored and the retention times were approximately 3.0 and 2.8 min for the drug and metabolite, together with their corresponding internal standards, respectively.

The assay limit of detection (LOD) was 1 ng/ml and lower limit of quantification (LLOQ) was 3 ng/ml. A freshly prepared stock of 10 µM ENZ (4,640 ng/ml) or 50 µM ENZ (23,200 ng/ml) was spiked into conditioned medium and diluted to a working concentration of 46.4 ng/ml or 232.0 ng/ml for use as a dilution quality control (DQC), to guarantee the integrity and accuracy of the test samples.

### Tissue preparation for MALDI imaging

PDEs were cultured in the absence or presence of ENZ for multiple time points as described above. At the time of harvest, explants were bisected along the marked tissue dye line that indicated the planar orientation of the tissue. One-half of the tissue was arranged in a cryo-mould filled with chilled 2% CMC and snap frozen. 10 µm sections of tissues were cut from the frozen block using a Shandon E cryotome (Thermo Scientific, Germany) and consecutive sections collected by thaw mounting on Superfrost glass slides (Thermo Scientific, Hungary) for haematoxylin and eosin (H&E) and drug imaging (MALDI MSI MS/MS). The other tissue halves were snap-frozen and stored at −80 °C for LC-MS/MS analysis as described above.

### Histology and immunohistochemistry

H&E slides were air-dried briefly (equilibrated to room temperature if frozen), heat–fixed on a heat block at 65 °C for 2 h and stained within 24 h according to an in-house protocol. Immunohistochemistry (IHC) slides were rinsed in Tris Buffered Saline (TBS) pH 7.6 and fixed in 4% paraformaldehyde (Sigma-Aldrich, NSW, Australia) at room temperature for 10 minutes. Slides were again rinsed with TBS for 5 minutes before they were placed on to the Discovery Ultra staining platform (Roche, Basel, Switzerland). Routine IHC was performed using the androgen receptor antibody (ab108341, Abcam, Cambridge, UK), anti-Rabbit HQ antibody (760-4815 Roche, Basel, Switzerland), anti-HQ HRP antibody (760-4820, Roche, Basel, Switzerland) and ChromoMap DAB kit (760-159, Roche, Basel, Switzerland) according to manufacturer’s instructions. Slides were counterstained with haematoxylin (760-2011, Roche, Basel, Switzerland) and bluing reagent (760-2037, Roche, Basel, Switzerland), dehydrated and mounted in DPX (LabChem, Zelienople, PA). Slides were scanned with an Axio Z.1 Scanner (Carl Zeiss Pty. Ltd., NSW, Australia) using an 40x objective, images were captured using Zen Blue 2.6 (Carl Zeiss Pty. Ltd., NSW, Australia) software.

### Matrix application on glass slide tissue sections

Application of matrix on tissue sections for MSI analyses was achieved by sublimation^15,45^. Slides stored at −80 °C were fully equilibrated to room temperature in a dry atmosphere to avoid condensation before processing began. Sublimation entailed depositing 300 µl of 10 mg/ml α-CHCA in methanol on the bottom of the glass chamber of the sublimation apparatus and allowing the solvent to evaporate to achieve a final dry weight of 3 mg of MALDI matrix. The sublimation device (Christ, John Morris Scientific) was connected to a vacuum pump, placed in an oil bath on a heating block and evacuated to a set pressure of 0.03 mbar. 100 ml of water was added to the water cooler of the sublimation apparatus, and a cylindrical ice block was suspended in the water without touching the bottom of the chamber and allowed to cool for 10 min. The heating block was set to 145 °C for at least 45 min. The matrix was gradually coated on the glass slide through a solid-gas phase state change because of the low pressure and high temperature difference in the closed system.

### MALDI MSI

MALDI MSI analysis was performed on PDEs treated with 10 µM ENZ (n = 1 patient) and 50 µM ENZ, (n = 2 patients; 3 PDEs). Tissue sections were analyzed on a MALDI SYNAPT HDMS Mass Spectrometer (Waters Corporation, Manchester, UK) operating in MS/MS imaging mode. Prior to analyses, the mass spectrometer was calibrated using a calibration solution of PEG and α-CHCA over the *m/z* range 50–990 Da in positive ion mode.

The *m/z* of the protonated precursor ion of ENZ is 465.10 Da. Enhanced duty cycle (EDC) mode was used to optimise the transmission of the dominant ENZ fragment ion m/z 209.09 (Supplementary Fig. S5). Scan time was set at 1.5 sec, 1 scan was recorded per pixel, laser settings used were repetition rate of 200 Hz and laser intensity was attenuated using a variable neutral density filter set to 150 a.u. The laser-raster step-size was set at 60 µm for both *x*- and *y*-directions with beam diameter of *ca* 100 µm.

The total ion count (TIC) normalisation method was used to determine intensity of a given m/z feature in the context of the entire dataset.

### Data processing and analysis

MALDI raw data files (.raw) were converted to MSI data files using HDImaging v1.4 software (Waters, Manchester, UK). The data processing settings were: resolution 8,000 full-width half-maximum (FWHM) and mass window 0.02 Da. The HDI imaging data was exported in continuum mode to the universal MSI file-sharing format- imzML. The imzML data was compatible for analysis in SCiLS Lab MVS (Bruker, Bremen, Germany). The peak of interest was the most abundant ENZ fragment ion of *m/z* 209.09 and was visualized for the six time points and normalised by TIC.

The LC-MS/MS data were processed using Analyst (Analyst 1.62, AB Sciex). The datafiles (.wiff and.scan files) were processed using a quantitation method tuned to measure the analyte and IS peak intensities at the expected elution times. The peak integration parameters were configured to a smoothing radius of 2 and bunching factor of 1–3. Automatic peak integration of all the peaks was manually checked and adjusted when necessary. The tabulated concentration for each QC samples was within 15% threshold (except for the LLOQ which is 20%). Interferences were assessed by including a biological matrix blank, unextracted IS solution and a spiked biological matrix of IS solution.

### Ethics approval and informed consent

Human ethics approval for this project was obtained from the Adelaide University Human Research Ethics Committee and the Research Ethics Committee of St Andrew’s Hospital (Adelaide, Australia). Fresh prostate cancer specimens were obtained with written informed consent through the Australian Prostate Cancer BioResource collection from men undergoing robotic radical prostatectomy at St Andrew’s Hospital. All experiments involving human tissue were performed in accordance with the relevant guidelines and regulations.

## Supplementary information

Supplementary information

## Data Availability

All data generated or analyzed during this study are either included in this published article (and its Supplementary Information Files) or are available from the Corresponding Authors upon request.
