# Supplementary material for: Evaluation of Small Molecule Drug Uptake in Patient-Derived Prostate Cancer Explants by Mass Spectrometry
Source: Sci Rep. 2019 Oct 18;9:15008. doi: 10.1038/s41598-019-51549-3 (PMC6802206; doi:10.1038/s41598-019-51549-3)
Supplement: Supplementary file 1 — Supplementary information [file 41598_2019_51549_MOESM1_ESM.docx]

**Evaluation of Small Molecule Drug Uptake in Patient-Derived Prostate Cancer Explants by Mass Spectrometry**

Shadrack M Mutuku^1,2,3^, Paul J Trim^3^, Bala K. Prabhala^3,4^, Swati Irani^1,2,5^, Kayla L Bremert^1,2,5^, Jessica M Logan^6^, Douglas A. Brooks^6^, Jürgen Stahl^7^, Margaret M Centenera^1,2,5^, Marten F Snel^3^, Lisa M Butler^1,2,5^

^1^Adelaide Medical School, University of Adelaide, Adelaide, SA 5005, Australia.

^2^Prostate Cancer Research Group, South Australian Health and Medical Research Institute (SAHMRI), SA 5000, Australia

^3^South Australian Health and Medical Research Institute (SAHMRI), Adelaide, SA 5000, Australia.

^4^Department of Drug Design and Pharmacology, University of Copenhagen, Denmark

^5^Freemasons Foundation Centre for Men’s Health, University of Adelaide, Adelaide, SA 5005, Australia.

^6^Mechanisms in Cell Biology and Disease Research Group, School of Pharmacy and Medical Sciences, Cancer Research Institute, University of South Australia, Adelaide, SA 5000, Australia

^7^ Clinpath Laboratories, Adelaide, SA 5000, Australia.

**Corresponding Authors:** Prof. Lisa Butler, Adelaide Medical School, University of Adelaide; Dr. Marten Snel, South Australian Health and Medical Research Institute, Adelaide SA 5000.

**Word count main text (excluding Abstract, Methods, References and figure legends):** 2,550 **Figures: 6 Tables: 1**

**Supplementary Information**

| **Patient** | **PDE label** | **Patient ID** | **Age RP** | **Pre RP PSA** | **P Stage** | **Gl (RP)** | **Gl (1°)** | **Gl (2°)** | **Core used for Research- Pathologist Rescore** |
| --- | --- | --- | --- | --- | --- | --- | --- | --- | --- |
| 1 | C | 33042R | 72.3 | 19.7 | PT3B | 7 | 4 | 3 | 6 (3+3) |
| 2 | D | 33036R | 71 | 2.24 | PT2C | 7 | 3 | 4 | Benign |
| 3 | E | 33050L | 69 | 18.3 | PT3A | 8 | 4 | 4 | 8 (4+4) |
| 4 | F | 33063L | 64.6 | - | PT3B | 9 | 4 | 5 | 9 (4+5) |
| 5 | G1 | 33055R | 73.4 | 18 | PT3A | 7 | 4 | 3 | Benign |
| 5 | G2 | 33055L | 73.4 | 18 | PT3A | - | - | - | Benign |
| 6 | H | 33026L | 59.6 | 5.5 | PT3A | 6 | 3 | 3 | Benign |
| 7 | J1 | 33061L | 69.8 | 4.6 | PT3A | - | - | - | 6 (3+3) |
| 7 | J2 | 33061R | 69.8 | 4.6 | PT3A | 7 | 4 | 3 | Benign |
| 8 | K1 | 33065L | 67.3 | 17 | PT3A | - | - | - | 6 (3+3) |
| 8 | K2 | 33065R | 67.3 | 17 | PT3A | 7 | 3 | 4 | Benign |

**Supplementary Table ST1. Clinical pathology of PDE tissues used in LC-MS/MS quantification assay.** Gl (RP)- represents overall Gleason score for the patient sample at the time of surgery by radical prostatectomy.

|  | |  | | **DMSO (ng/ml)** | | | | | | | | | | **ENZ (ng/ml)** | | | | | | | | | |
| --- | --- | --- | --- | --- | --- | --- | --- | --- | --- | --- | --- | --- | --- | --- | --- | --- | --- | --- | --- | --- | --- | --- | --- |
|  |  |  |  | **0 h** | | **48 h** | | | | **0 h** | **48 h** | **0 h**  **(50µM)** | **48 h**  **(50µM)** | **0 h** | | **48 h** | | | | **0 h** | **48 h** | **0 h**  **(50µM)** | **48 h**  **(50µM)** |
| **Patient** | **PDE** | **DQC** | **Accuracy** | **DS** | **DQ** | **DSS1** | **DSS2** | **DQS1** | **DQS2** | **MALDI** | **MALDI** | **MALDI** | **MALDI** | **DS** | **DQ** | **DSS1** | **DSS2** | **DQS1** | **DQS2** | **MALDI** | **MALDI** | **MALDI** | **MALDI** |
| 1. | C | 45.8 | 98.7% | BLOQ | BLOQ | BLOQ | BLOQ | BLOQ | BLOQ | BLOQ | BLOQ | N/A | N/A | 5,650 | 4,040 | 5,270 | 5,330 | 4,420 | 4,530 | 4,890 | 4,640 | N/A | N/A |
| 2. | D | 45.8 | 98.7% | BLOQ | BLOQ | BLOQ | BLOQ | BLOQ | BLOQ | BLOQ | BLOQ | N/A | N/A | 4,950 | 5,090 | 4,240 | 4,910 | 5,200 | 5,690 | 5,260 | 5,250 | N/A | N/A |
| 3. | E | 53.1 | 114.4% | BLOQ | BLOQ | BLOQ | BLOQ | BLOQ | BLOQ | BLOQ | BLOQ | N/A | N/A | 6,230 | 4,520 | 5,060 | 4,800 | 4,150 | 4,450 | 4,240 | 4,640 | N/A | N/A |
| 4. | F | 53.1 | 114.4% | BLOQ | BLOQ | BLOQ | BLOQ | BLOQ | BLOQ | BLOQ | BLOQ | N/A | N/A | 4,360 | 5,030 | 4,280 | 4,610 | 4,740 | 4,980 | 4,730 | 5,250 | N/A | N/A |
| 5. | G1 | 51.3 | 110.5% | BLOQ | BLOQ | BLOQ | BLOQ | BLOQ | BLOQ | BLOQ | BLOQ | N/A | N/A | 5,320 | 5,520 | 4,550 | 4,270 | 3,970 | 3,720 | 5,310 | 4,510 | N/A | N/A |
| 5. | G2 | 51.3 | 110.5% | BLOQ | BLOQ | BLOQ | BLOQ | BLOQ | BLOQ | BLOQ | BLOQ | N/A | N/A | 4,620 | 4,080 | 3,950 | 4,270 | 3,700 | 3,880 | 5,170 | 4,010 | N/A | N/A |
| 6. | H | 51.3 | 110.5% | BLOQ | BLOQ | BLOQ | BLOQ | BLOQ | BLOQ | BLOQ | BLOQ | BLOQ | BLOQ | 4,950 | 4,800 | 4,520 | 4,490 | 4,850 | 5,110 | 5,460 | 4,400 | 23,100 | 22,000 |
| 7. | J1 | 44.7 | 96.4% | BLOQ | BLOQ | BLOQ | BLOQ | BLOQ | BLOQ | BLOQ | BLOQ | BLOQ | BLOQ | 4,740 | 4,120 | 4,010 | 4,410 | 4,020 | 4,230 | 4,890 | 4,110 | 20,300 | 20,700 |
| 7. | J2 | 44.7 | 96.4% | BLOQ | BLOQ | BLOQ | BLOQ | BLOQ | BLOQ | BLOQ | BLOQ | BLOQ | BLOQ | 4,490 | 5,340 | 5,000 | 4,690 | 4,680 | 4,640 | 4,920 | 4,930 | 21,600 | 20,700 |
| 8. | K1 | 42.9 | 92.4% | BLOQ | BLOQ | BLOQ | BLOQ | BLOQ | BLOQ | BLOQ | BLOQ | BLOQ | BLOQ | 4,880 | 4,870 | 4,220 | 4,520 | 4,700 | 4,840 | 4,230 | 3,820 | 22,700 | 20,500 |
| 8. | K2 | 42.9 | 92.4% | BLOQ | BLOQ | BLOQ | BLOQ | BLOQ | BLOQ | BLOQ | BLOQ | BLOQ | BLOQ | 4,720 | 4,370 | 4,470 | 4,440 | 4,180 | 4,110 | 4,170 | 3,840 | 20,100 | 19,500 |

**Supplementary Table ST2. Summary data for LC-MS/MS quantification of ENZ in M4 media**. PDEs from n=8 patients (11 PDEs) were cultured in medium containing 10 µM ENZ or DMSO control over 48 h. Prior to incubation, at 0 h, cultured media was collected for drug stability (DS – medium with gelatin sponge only) and drug quantification (DQ – medium with explant atop of a gelatin sponge). Quadruplicate PDE tissues were placed evenly in separate DQ wells, DQS1 and DQS2 whilst DS wells, DSS1 and DSS2, monitored integrity of ENZ over the 48 h incubation period. An extra subset of tissues from n=3 patients were cultured in medium containing 50 µM ENZ or DMSO control over 48 h for MALDI MSI. Dilution quality control (DQC) indicates a freshly prepared stock of 10 µM ENZ (4,640 ng/ml) or 50 µM ENZ (23,200 ng/ml) was spiked into conditioned medium and diluted to a working concentration of 46.4 ng/ml or 232.0 ng/ml. BLOQ - below LLOQ.

|  | **Measured Concentration (ng)** | | | | | | | | | | **Normalised (ng/µg) Total Protein** | | | | | | | | |
| --- | --- | --- | --- | --- | --- | --- | --- | --- | --- | --- | --- | --- | --- | --- | --- | --- | --- | --- | --- |
|  | **Day Ø** | **DMSO, control** | | | | | **ENZ, 10 µM treatment** | | | | **Day Ø** | **DMSO, control** | | | | **ENZ, 10 µM treatment** | | | |
| **Patient** | **PDE** | **QC** | **QC/1** | **2** | **3** | **4** | **5** | **6** | **7** | **8** | **QC** | **QC/1** | **2** | **3** | **4** | **5** | **6** | **7** | **8** |
| 1. | C | _ | 0.00 | 0.00 | 0.00 | 0.00 | 4.13 | 3.68 | 2.62 | 2.17 | _ | 0.00 | 0.00 | 0.00 | 0.00 | 0.44 | 0.36 | 0.49 | 0.40 |
| 2. | D | 0.14 | 0.00 | 0.00 | 0.00 | 0.00 | 5.84 | 4.78 | 6.74 | 6.18 | 0.01 | 0.00 | 0.00 | 0.00 | 0.00 | 0.31 | 0.33 | 0.32 | 0.47 |
| 3. | E | 0.00 | 0.00 | 0.00 | 0.00 | 0.00 | 3.52 | 2.89 | 4.41 | 7.31 | 0.00 | 0.00 | 0.00 | 0.00 | 0.00 | 0.22 | 0.30 | 0.20 | 0.24 |
| 4. | F | 0.01 | 0.00 | 0.00 | 0.00 | 0.00 | 4.6 | 2.21 | 5.47 | 4.15 | 0.00 | 0.00 | 0.00 | 0.00 | 0.00 | 0.46 | 0.55 | 0.44 | 0.26 |
| 5. | G1 | 0.01 | 0.00 | 0.00 | 0.00 | 0.00 | 2.09 | 1.78 | 2.99 | 2.18 | 0.00 | 0.00 | 0.00 | 0.00 | 0.00 | 0.33 | 0.29 | 0.40 | 0.43 |
| 5. | G2 | 0.02 | 0.00 | 0.00 | 0.00 | 0.00 | 1.16 | 3.8 | 1.24 | 2.25 | 0.00 | 0.00 | 0.00 | 0.00 | 0.00 | 0.42 | **1.39* | 0.48 | 0.36 |
| 6. | H | 0.00 | 0.00 | 0.00 | 0.00 | 0.00 | 5.53 | 4.77 | 7.67 | 7.98 | 0.00 | 0.00 | 0.00 | 0.00 | 0.00 | 0.37 | 0.35 | 0.47 | 0.64 |
| 7. | J1 | 0.00 | 0.00 | 0.00 | 0.00 | 0.00 | 3.48 | 3.70 | 4.51 | 4.22 | 0.00 | 0.00 | 0.00 | _ | 0.00 | 0.30 | 0.26 | 0.31 | 0.29 |
| 7. | J2 | 0.00 | 0.00 | 0.00 | 0.00 | 0.00 | 5.48 | 9.51 | *4.18* | 8.02 | 0.00 | 0.00 | 0.00 | 0.00 | 0.00 | 0.35 | 0.62 | 0.43 | 0.60 |
| 8. | K1 | 0.00 | 0.00 | 0.00 | 0.00 | 0.00 | 11.7 | 5.99 | 9.93 | 12.5 | 0.00 | 0.00 | 0.00 | 0.00 | 0.00 | 0.36 | 0.30 | 0.35 | 0.42 |
| 8. | K2 | 0.13 | 0.00 | 0.00 | 0.00 | 0.00 | 14.6 | 6.11 | 9.08 | 10.6 | 0.00 | 0.00 | 0.00 | 0.00 | 0.00 | 0.31 | 0.30 | 0.37 | 0.34 |

**Supplementary Table ST3. Summary data for LC-MS/MS quantification of ENZ in PDE homogenates.** PDEs from n=8 patients (11 PDEs) were cultured in medium containing 10 µM ENZ or DMSO control over 48 h as previously outlined ([Supplementary Table ST](#SuppTable1)2). Intra-patient variability is the percent average coefficient of variation (%) of measured drug concentration (ng/µg) in replicate PDEs from each patient. Inter-patient variability is the percent average of the mean drug concentration (ng/µg) across the entire patients. The drug absorption had an intra- and inter-patient variability of 17.7% and 20.1%, respectively. Values in italics not used in calculation.

**
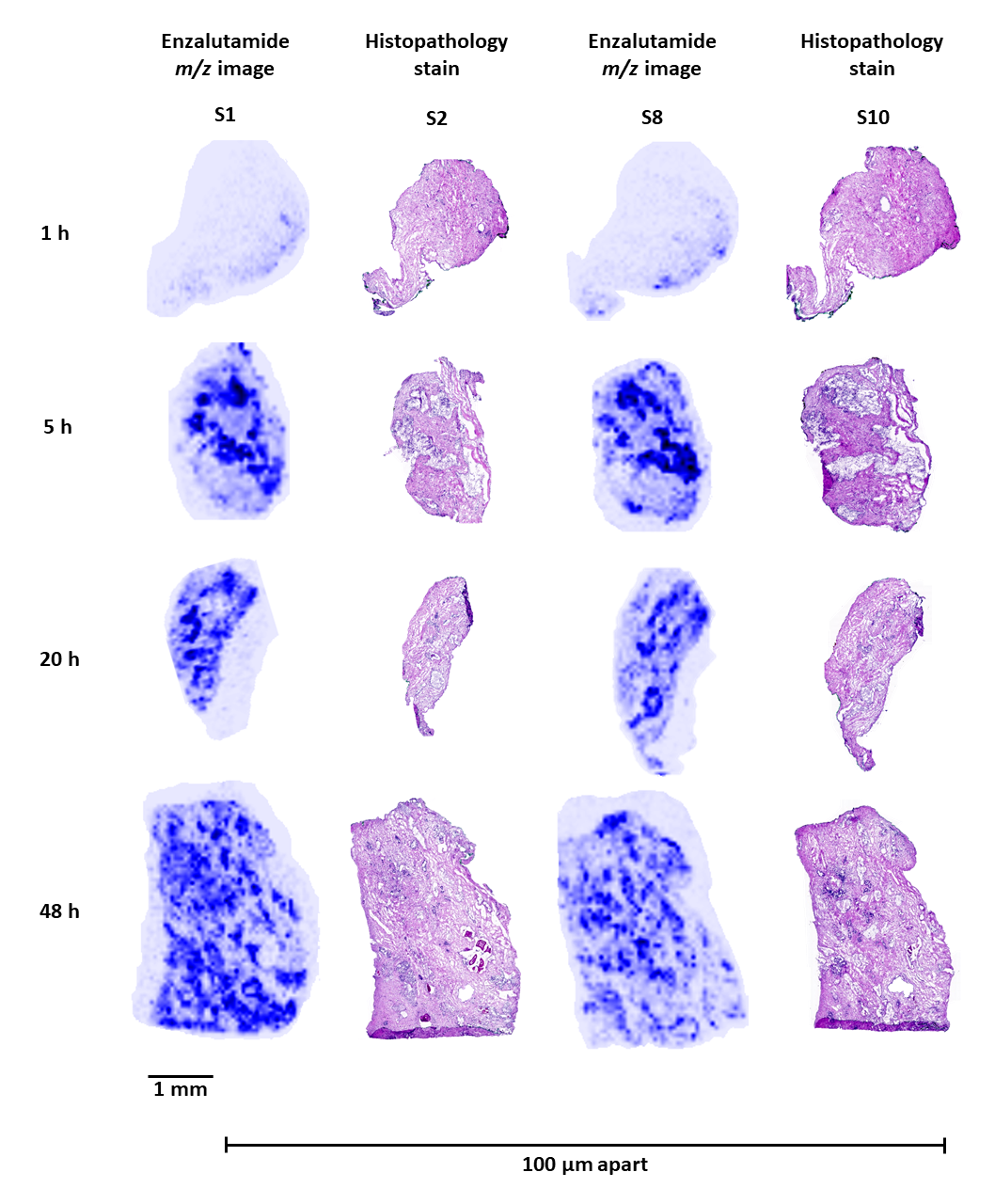
Supplementary Figure S1. Time profile of** **ENZ penetration in *ex vivo* prostate tissue culture**. **A**. Four PDE’s from a single patient were cultured in medium containing 50-µM ENZ over a 48-h period. MALDI MSI MS/MS images (S1 and S8) normalised to TIC of the predominant ENZ fragment ion, *m/z* 209.09, next to a H&E stained serial sections (S2 and S10) at 0 h, 5 h, 20 h and 48 h. S1 and S2 are adjacent sections whereas S8 and S10 are 10 µm apart; S1 and S10 represent a cryo-sectioning depth of 100 µm into the PDE block.

**
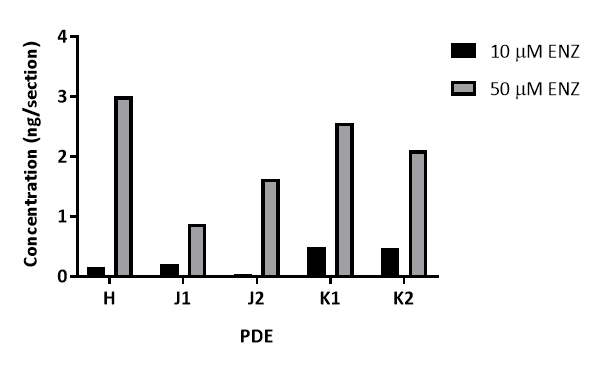
**

**Supplementary Figure S2**. **LC-MS/MS quantification of ENZ in PDE homogenates derived from thin cryosections**. PDEs from different patients were cultured in medium containing 10 µM ENZ or 50 µM ENZ over a 48-h period and harvested by embedding in 2% CMC. Samples were cryo-sectioned at a thickness of 10 µm. For equivalent dosage, n=5 section(s) (10 µM ENZ), dark grey bar and n=1 section(s) (50 µM ENZ) light grey bar, were collected and analysed by LC-MS/MS as earlier described. Data displayed as ng per tissue section. Sample volume was insufficient for total protein measurements.

**
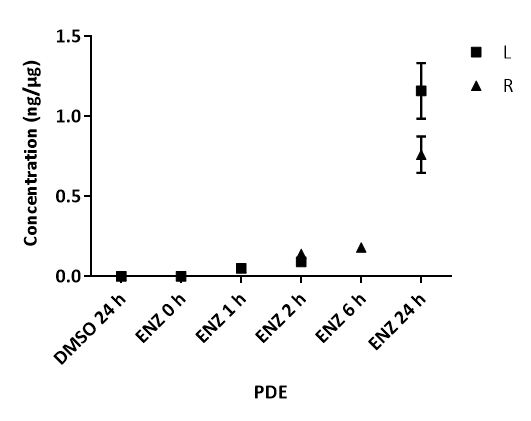
**

**Supplementary Figure S3. LC-MS/MS quantification of ENZ in PDE homogenates over a time course.** Six PDEs from either the left (L) and right (R) prostate cores were cultured in 10 µM ENZ and harvested at 0 h, 1 h, 2 h, 6 h, 24 h or 24 h (DMSO control) and bisected into two halves. One-half was homogenised as described for LC-MS/MS analysis. Drug concentration is normalised to total protein expressed as ng/µg. *Note: PDE for L at 6 h lost during sample homogenisation**.** Error bars indicate the 15% CV threshold allowed for accuracy and precision of assay.

**
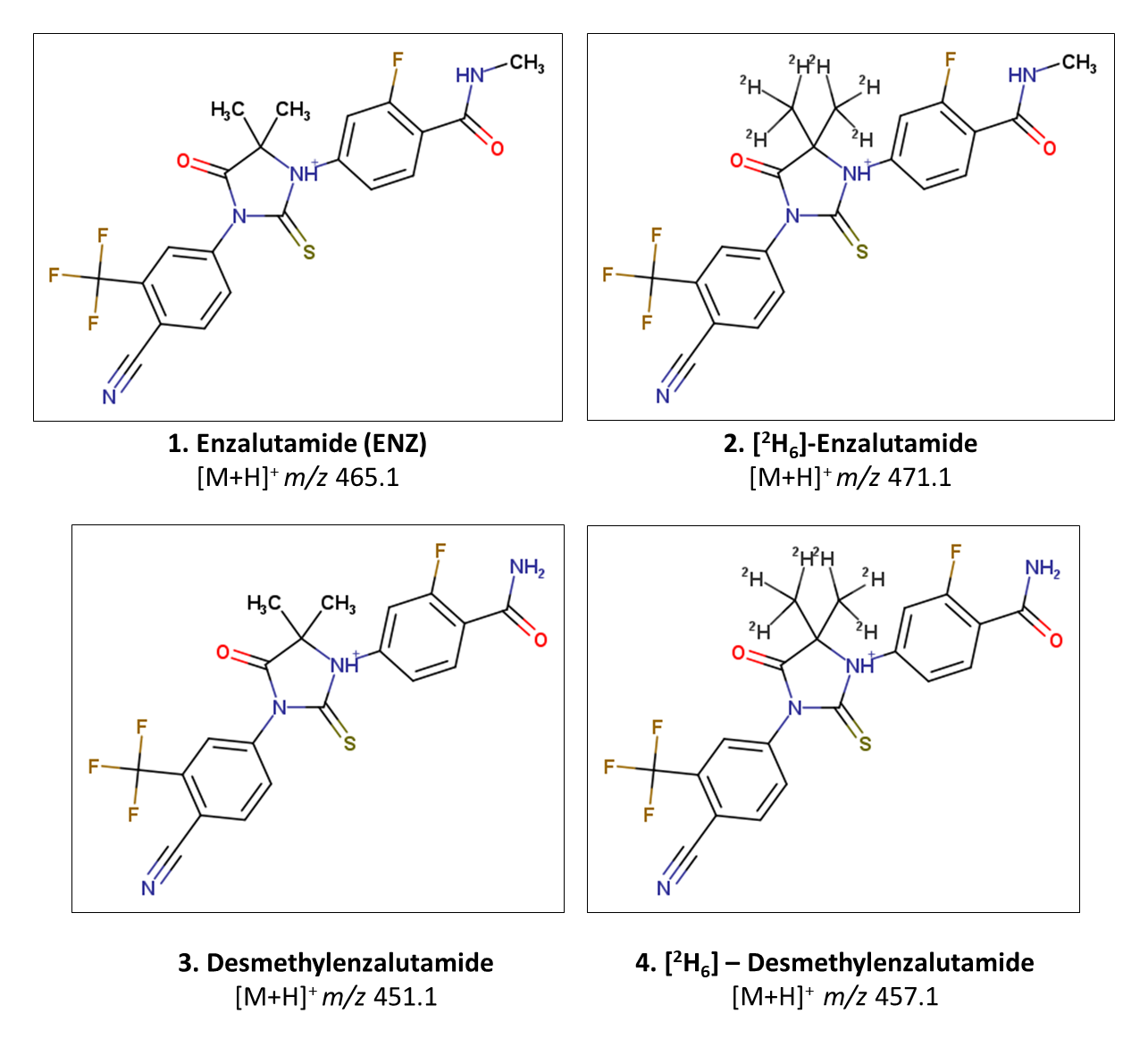
**

**Supplementary Figure S4. Chemical structures**. Molecular structures and exact molecular weights for MRM selection of **1.** enzalutamide (ENZ), **2.** deuterated ENZ, **3.** desmethylENZ and **4.** deuterated desmethylENZ. Structures drawn using MarvinSketch version 18.19.0.

**
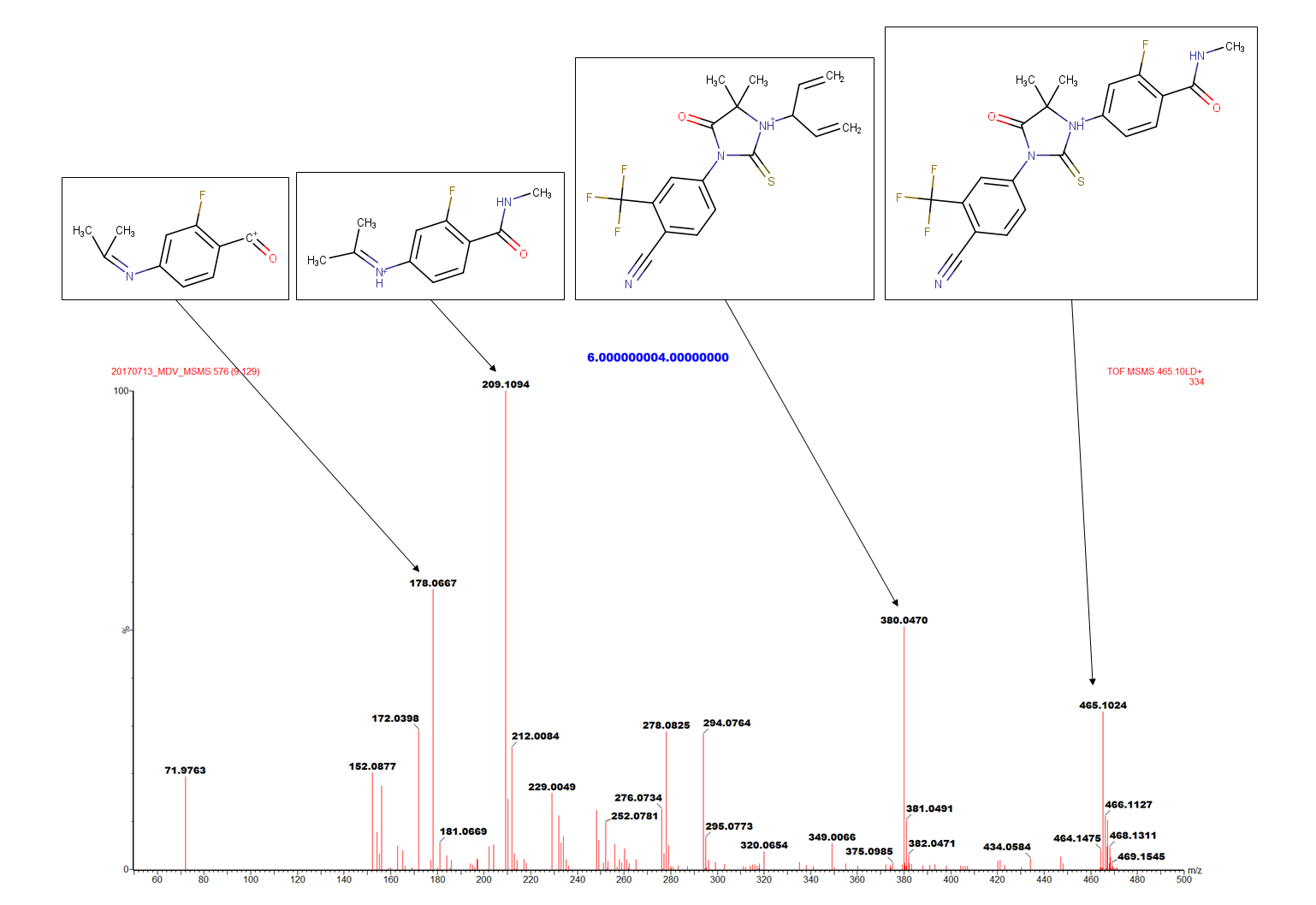
**

**Supplementary Figure S5. MRM transmission of fragment ions of enzalutamide in positive ion mode MALDI MSI**. *m/z* 209.1 is the most dominant fragment ion of the collision induced dissociation (CID) of the precursor *m/z* 465.1. Other confirmation ions are *m/z* 380.0 and *m/z* 178.0.

**Supplementary Table ST1. Clinical pathological assessment of Day 0 formalin-fixed paraffin-embedded samples of PDE tissues used in LC-MS/MS quantification assay.** Gl (RP)- represents overall Gleason score for the patient sample at the time of surgery by radical prostatectomy.

**Supplementary Table ST2. Summary data for LC-MS/MS quantification of enzalutamide in M4 media**. PDEs from n=8 patients (11 PDEs) were cultured in medium containing 10 µM ENZ or DMSO control over 48 h. Prior to incubation, at 0 h, cultured media was collected for drug stability (DS – medium with gelatin sponge only) and drug quantification (DQ – medium with explant atop of a gelatin sponge). Quadruplicate PDE tissues were placed evenly in separate DQ wells, DQS1 and DQS2 whilst DS wells, DSS1 and DSS2, monitored integrity of ENZ over the 48-h incubation period. An extra subset of tissues from n=3 patients were cultured in medium containing 50 µM ENZ or DMSO control over 48 h for MALDI MSI. Dilution quality control (DQC) indicates a freshly prepared stock of 10 µM ENZ (4,640 ng/ml) or 50 µM ENZ (23,200 ng/ml) was spiked into conditioned medium and diluted to a working concentration of 46.4 ng/ml or 232.0 ng/ml. BLOQ - below LLOQ.

**Supplementary Table ST3. Summary data for LC-MS/MS quantification of enzalutamide in PDE homogenates.** PDEs from n=8 patients (11 PDEs) were cultured in medium containing 10 µM ENZ or DMSO control over 48 h as previously outlined ([Supplementary Table ST](#SuppTable1)2). Intra-patient variability is the percent average coefficient of variation (%) of measured drug concentration (ng/µg) in replicate PDEs from each patient. Inter-patient variability is the percent average of the mean drug concentration (ng/µg) across the entire patients. The drug absorption had an intra- and inter-patient variability of 17.7% and 20.1%, respectively. Values in italics not used in calculation.
